# Prescribing errors in electronic prescriptions for outpatients intercepted by pharmacists and the impact of prescribing workload on error rate in a Chinese tertiary-care women and children’s hospital

**DOI:** 10.1186/s12913-019-4843-1

**Published:** 2019-12-30

**Authors:** Jian-hui Yang, Yu-fang Liao, Wu-bin Lin, Wen Wu

**Affiliations:** Department of Pharmacy, Women and Children’s Hospital, School of Medicine, Xiamen University, No. 10 Zhenhai Road, Xiamen, 361001 China

**Keywords:** Outpatients, Pharmacist-led intervention, Prescribing errors, Prescribing workloads, Subgroup analysis, Time-series analysis, China

## Abstract

**Background:**

Prescribing errors may, influenced by some risk factors, cause adverse drug events. Most studies in this field focus on errors in prescriptions for hospital inpatients, with only a few on those for outpatients. Our study aimed to explore the incidence of prescribing errors in electronic prescriptions and illustrate the trend of prescribing workload and error rate over time.

**Methods:**

The cross-section study was performed between September, 2015 and November, 2015. Prescribing errors were intercepted by pharmacists using a prescription reviewing system under which prescriptions with errors were transferred to a specific computer and recorded by another pharmacist and the incidence of total prescribing errors and severe errors was then calculated. A subgroup analysis was conducted in accordance to the number of drug orders, the age group of patients, the seniority of physicians, the specialty of physicians, the working day when prescriptions were issued, and the prescribing workload of physicians. A time-series analysis was employed to analyze the trend of prescribing workload and error rate, and the correlation between them.

**Results:**

Totally, 65,407 patients were included in this study and 150,611 prescriptions with 294,564 drug orders (including 584 different drugs) were reviewed for identification of errors. A total of 534 prescribing errors (an error rate of 0.34%) were identified. Severe errors accounted for 13.62% of total errors. The subgroup analysis showed prescriptions of multiple drug orders, for pediatric patients aged 29 days to 12 years, from physicians specializing in ophthalmology and otorhinolaryngology, or prescribing on weekdays were more susceptible to errors. A time-series analysis demonstrated no correlation between prescribing workload and error rate which increased at the end of each working shift while prescribing workload decreased.

**Conclusion:**

Less than 1% of the studied prescriptions came with errors among which one in seven were severe ones. But prescribing errors were in no relation to workloads. What’s more, further studies are needed to investigate pharmacist-led intervention to reduce prescribing errors.

## Background

Medication errors are associated with a large number of deaths in UK and the USA, with estimated annual mortality of 712 and 98,000 respectively [1, 2], thus preventing errors may improve patient outcomes. Medication errors refer to errors occurring during medication prescribing, transcribing, dispensing, administering, or monitoring [3]. As an important part of medication errors, prescribing errors occur during the process of decision making and prescription writing by physicians [4], which may cause adverse drug events (ADEs) [5], and accordingly lead to health-associated costs [6, 7] and an excess length of stay [7]. It has been found that an ADE will extend a stay for 1.91 more days, along with an extra cost of $2262 [7].

Females, especially pregnant women, and children are very susceptible to ADEs due to prescribing errors. Obstetricians usually face a challenge in judging the teratogenicity of a drug and its potential harm to the fetus in pregnant women [8]. On the other hand, the dose for children is based on their body weight, which is more than likely to result in wrong calculations. Besides, children are unable to elaborate their feelings clearly to physicians after a prescribing error occurred. More importantly, children are less tolerant of ADEs than adults due to their premature organs [3].

Many approaches have been used to reduce prescribing errors. Electronic prescribing is an effective technology-based strategy, which can, compared with hand-written prescribing, largely reduce errors [9]. However, despite introduction of improved electronic technologies, prescribing errors still exist and have presented some new types [10]. Pharmacist-led intervention also plays a significant role in reducing prescribing errors with those technologies [11–13]. Patients, with the pharmacist-led intervention, were found less likely to be prescribed a non­selective NSAID, β blocker, ACE inhibitor or loop diuretic when they were under the medical condition of peptic ulcer without gastroprotection, asthma, pregnancy or failure of appropriate monitoring respectively [11]. In our study, pharmacists were proved to be an essential role in medical treatment since all of the prescribing errors identified were intercepted by them.

Preventive measures are also effective methods for reducing a range of errors and require a better understanding of complex factors that contribute to those errors [14]. Some risk factors associated with prescribing errors made by physicians had been identified. Working conditions of physicians, including stress [15], prescribing workload [16, 17], interruption [18] and fatigue [19], were considered to contribute substantively to this issue. In addition, the mental conditions of physicians such as depression had also been studied, and it was revealed that such mental disorder was likely to cause more errors [19, 20]. In view of the fact that most studies only examined the relationship between prescribing workload and error rate by chi square test or logistic regression without introducing time variable, we focused our study on the trend of prescribing workload and error rate over time.

The mode of Hospital Information System (HIS)-assistant prescription reviewing and feedback before medication dispensing was not prevalent in Chinese medical institutions during the last decades. Most prescription assessments were conducted at the end of an index month or quarter when prescribing errors had occurred before identified. In June 2018, the National Health Commission of the People’s Republic of China issued a policy called *Standards for Prescription Reviewing in Medical Institutions* [21], which stipulates that all prescriptions should be reviewed prior to prescription pricing and medication dispensing. Before the policy was launched, pharmacists in our hospital had already been practicing the mode of prescription reviewing with the assistance of an electronic system for several years, so many prescribing errors had been intercepted before reaching patients to whom potential harms were thus prevented.

Compared with other study [22] which just investigated a fraction of prescriptions due to the investigating method of surveys or chart reviews, our study included all outpatient e-prescriptions issued by physicians in our hospital during the study period. To our knowledge, few studies on prescription reviewing analyzed prescribing errors before they occurred in outpatients in China, and explored the correlation between prescribing workload and error rate at a specific time. Therefore, our study aimed to investigate the prevalence of prescribing error in women and children and the relationship between prescribing workload and error rate.

## Methods

### Setting and study population

This cross-section study was conducted between September, 2015 and November, 2015 in an outpatient setting at a tertiary-care teaching hospital with 1100 staff, 700 beds, and 1.4 million annual outpatient visits, rather than a community-based setting. The major outpatient clinics in our hospital include clinics concerning obstetrics, gynecology, pediatrics, family planning and reproductive medicine.

Our hospital mainly treats adult female and pediatric patients. The outpatient service involving a total of 228 physicians is provided normally from 8:00 to 12:00 and 14:30 to 17:30, with a delay of less than an hour at both shifts.

### Hospital information system (HIS) for drug-order prescribing and prescription reviewing service

The system was developed by ZOE SOFT Corp. and brought into operation in 2004. It includes two electronic prescription subsystems for physician prescribing and pharmacist reviewing respectively. The prescribing system requires physicians to enter all components contained in a prescription and at least one indication and one medication, and offers only basic prescribing service without mandatory default dose, frequency, route and automatic checks for errors.

### Prescribing error reviewing

A pharmacist team including twenty members work on prescription reviewing. All the pharmacists were examined and qualified after a uniform training for error detecting. Prior to medication dispensing, prescriptions for adults and children were reviewed separately through a prescription reviewing system by two pharmacists each after drug orders were approved by physicians. This was a routine check by the pharmacists during their shift. In case of tiredness, the reviewing pharmacists changed shift every hour. Each pharmacist reviewed prescriptions independently with their own expertise after a uniform training, and the assessment of their qualification had been conducted before (The test score of prescribing-error detecting should be no less than 90 for qualification.).

The details of prescription reviewing comprise patient conditions (age, gender and diagnosis) and therapy regimens (medication selection, dose, frequency, route of administration and drug-drug interaction). Drugs are categorized according to the organ or system on which the drugs act, based on the adaptation of Anatomical Therapeutic Chemical (ATC) classification [23], which adds Chinese patent medicines into the classification items.

**Fig. 1:**
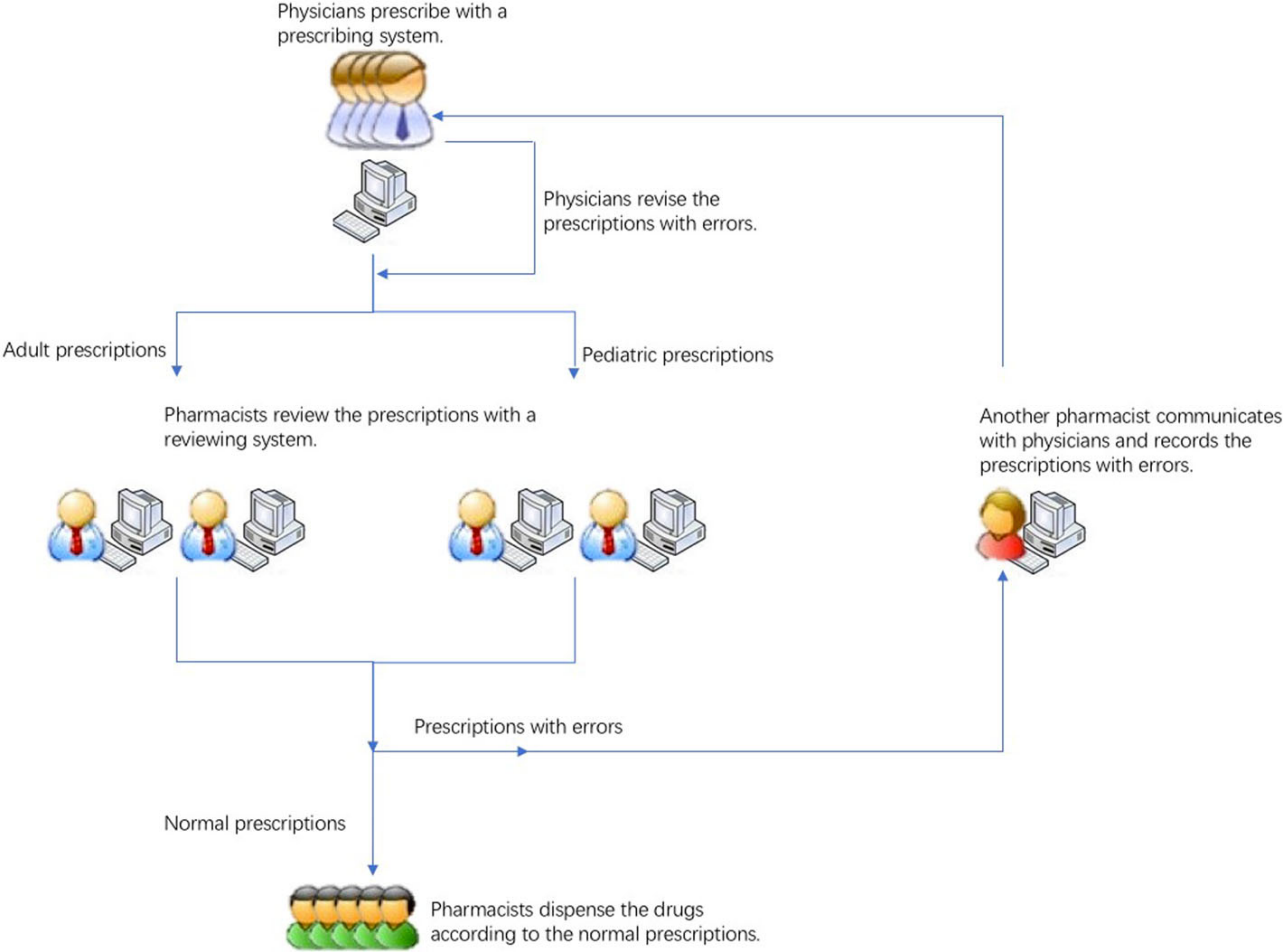
The workflow of pharmacists intercepting prescribing errors

If a prescription with errors was identified by a pharmacist, it would be transferred to a specific computer. Another pharmacist validated the prescribing error again, and subsequently called the prescribing physician to correct the wrong prescription and, meanwhile, documented the errors. The workflow is shown in Fig. 1.

All documented prescriptions with errors were reviewed by investigators and included for analysis during the study period.

### Classification of prescribing errors

Medication prescribing errors are defined as a deviation from drug labels or approved off-label use in accordance with practice guidelines in our hospital. They are classified according to an adaptation of *NCC MERP Taxonomy of Medication Errors* issued by the National Coordinating Council for Medication Error Reporting and Prevention (NCCMERP) [24]. The adaptation was required in our study for the classification of prescribing errors due to the introduction of the electronic prescribing system and the nature of the outpatient setting. The errors were classified into incomplete prescribing and incorrect prescribing. Incomplete prescribing was defined as the omission of components from a normal prescription, including the omission of other diagnoses. The omission of drugs was excluded because the patients might have possessed the indicated medication at home or their certain condition need no pharmaceutical therapy. Incorrect prescribing involved improper dose (exceeding a 20% tolerance), wrong frequency, wrong route of administration, wrong drugs, wrong diagnosis, wrong strength, wrong dosage form, deteriorated drug errors, and monitoring errors (including adverse drug-drug interaction and contraindication). Dose omission, wrong technique, wrong rate, wrong duration and wrong patient, which were included as medication errors in *NCC MERP Taxonomy of Medication Errors*, were all excluded from our study because those types of errors could not be determined from the prescriptions for outpatients.

### Determination of the severity of prescribing errors

The severity of errors were determined independently by two investigators, according to an adaptation of standards set up by the NCCMERP [25] which were based on the assessment of outcomes for the patients receiving errors. Since all the errors had been intercepted by pharmacists in our study, the assessment of severity was based on the hypothesis that errors had reached patients. The level of severity ranked from Category A to I in an ascending order. The disparity or doubts in determination was resolved through discussions with a third investigator. The errors at a severity level from D to I were classified as severe errors because they could cause potential injuries.

### Control group

As a control group, normal prescriptions without errors were extracted from the Hospital Information System and saved meanwhile in an Excel form of which the data includes patient demographics, diagnoses, and details of medication use.

### Subgroup analysis of errors

The subgroup analysis of prescribing errors was conducted according to the number of drug orders, the age group of patients, the seniority of physicians, the specialty of physicians, the working day when prescriptions were issued and the workload of physicians.

The number of drug orders refers to the number of orders at each prescription instead of the total number of drugs a patient was taking at the time.

Age grouping was based on the grouping method in *Pediatric & Neonatal Dosage Handbook* [26]. All age groups included neonate (~ 28 days old), infant (29 days to 12 months old), child (1 to 12 years old), adolescent (13 to 18 years old) and adult (> 18 years old).

The seniority of physicians was divided into three levels, namely, junior level, intermediate level and senior level. Junior physicians are those who have received a qualification certificate of physicians. Intermediate physicians are those who have received an intermediate certificate and worked for more than 4 years since becoming a junior physician. Senior physicians are those who have received a senior certificate and worked for more than 5 years since becoming an intermediate physician.

The specialty of physicians included obstetrics, pediatrics, gynecology, family planning, reproductive medicine, dermatology, general surgery, general medicine, ophthalmology and otorhinolaryngology.

The working days were divided into weekdays (Monday to Friday) and weekends (Saturday and Sunday).

Our study calculated the workload at each time interval and rated workloads with two levels, namely, high workloads and low workloads, in accordance to the median value of workload at each time interval.

### Statistical analysis

Descriptive statistics were expressed as mean ± standard deviation in continuous data (non-normal distribution data expressed as median with interquartile range). Categorical data were presented as percentage rates.

The level of agreement on error severity between investigators was measured by kappa coefficient.

The subgroup analysis was performed through univariate logistics regression.

The trend of prescribing workload and error rate over time and the association between them were analyzed using time-series analysis. The model type was determined by the time-series plot, autocorrelation function and partial autocorrelation function. We set the workload as independent variable and error rate as dependent variable in the model, calculated the coefficient (*β* value) of the independent variable (workload) and determined whether its value was statistically significant for determining the association between those two variables. The *β* value indicates the error rate changes when workload changes by one unit within a time interval.

The shift was divided by a 15-min interval from 8:00–8:15 to 17:30–17:45. The time interval was set as 15 min which exceeded the average time for each physician to issue a prescription, because we wanted to draw as many time points as possible to figure out an accurate trend of prescribing workloads over time.

The workload was the average number of prescriptions issued by one physician in a certain time slot over a 91-day period, calculated by the following formula: Set *N* = total number of prescriptions / total number of physicians in a certain time slot (e.g. 8:00–8:15), then workload (e.g. 8:00–8:15) = [*N*_*1*_ (2015-09-01 8:00–8:15) + *N*_*2*_ (2015-09-02 8:00–8:15) + … … + *N*_*91*_(2015-11-30 8:00–8:15)]/91 days. The error rate was the percentage of prescriptions with errors in a certain time slot over a 91-day period, calculated by the following formula: Set *n* = number of prescriptions with errors at a time interval (15 mins), *m* = total number of prescriptions in a certain time slot, then error rate (e.g. 8:00–8:15) = [*n*_*1*_ (2015-9-1 8:00–8:15) + *n*_*2*_ (2015-9-2 8:00–8:15) + …… + *n*_*91*_ (2015-11-30 8:00–8:15)]/[*m*_*1*_ (2015-9-1 8:00–8:15) + *m*_*2*_ (2015-9-2 8,00–8:15) + … + *m*_*91*_ (2015-11-30 8:00–8:15)] * 100%. The above formulas were applied to the calculation of workloads and error rates in other time slots as well.

The SPSS software (IBM Corp, version 23) was used for all statistical analyses. *P* value less than 0.05 was set as threshold for statistical significance.

## Results

### Overview of included prescriptions for outpatients

During the 3-month period, a total of 65,407 patients were studied. The median age of adults was 30 years old (interquartile 26–35 years old) while that of children was 1 year old (interquartile 0.58–3 years old). 150,611 prescriptions overall with 294,564 drug orders including 584 different drugs were reviewed by pharmacists before drug dispensing. Pediatric prescriptions accounted for 43.33% of all (65,259 out of 150,611 prescriptions). The drug orders in each prescription averaged 1.96, ranging from 1 to 10. The most prescribed drugs were desloratadine for suspension (4.14% of all prescriptions), budesonide suspension for inhalation (2.81%) and a compound of ambroxol hydrochloride and clenbuterol hydrochloride oral solution (2.46%). Infertility (7.19% of all prescriptions) and pregnancy supervision (6.11%) were the most common diagnoses among adult patients while acute upper respiratory infection (6.95%) and acute bronchitis (6.64%) were among pediatrics. An average of approximately 60 prescriptions were reviewed by a pharmacist during a one-hour interval.

### Analysis of prescribing errors

510 prescriptions were identified with a total of 534 errors, with 24 prescriptions having 2 errors and none having 3 or more errors. The most errors were incomplete prescribing due to the omission of secondary diagnoses (251 out of 534 errors, 47.00%), followed by incorrect prescribing including improper dose (135, 25.28%), wrong frequency (45, 8.43%), wrong diagnosis (36, 6.74%), wrong route of administration (33, 6.18%) and other types of errors. The detail of each type of errors were also shown at Table 1. The 3 most common medication categories in wrong prescriptions were anti-infective drugs for systemic use, drugs for the alimentary tract and metabolism, and Chinese patent medicines, accounting for 35.02, 21.72 and 10.11% of all errors respectively.

**Table 1:** Category and detail of prescribing errors

Category	Type of errors (*n*_0_, %)	Detail of errors (*n/n*_0_, %')
Incomplete	Omission of other diagnosis^a^ (251, 47.00%)	Top 3
		Vaginitis (89/251, 35.46%)
		Acute upper respiratory tract infection (36/251, 14.34%)
		Mastitis (10/251, 3.98%)
	Omission of other drugs	Not applicable
Incorrect	Improper dose (135, 25.28%)	Overdose (typing errors^b^) (48/135, 35.56%)
		Underdose (typing errors) (35/135, 25.93%)
		Wrong unit leading to wrong dose (typing errors) (32/135, 23.70%)
		Underdose (conscious act^c^) (11/135, 8.15%)
		Overdose (conscious act) (10/135, 7.41%)
	Wrong frequency (45, 8.43%)	Top 3
		‘Bid^d^’ prescribed wrong as ‘Qd’ (19/45, 42.22%) eg. Cefuroxime, Doxycycline
		‘Qd’ prescribed wrong as ‘Tid’ (5/45, 11.1%) eg. Azithromycin
		‘Qd’ prescribed wrong as ‘Bid’ (4/45, 8.89%) eg. Desloratadine
	Wrong diagnosis (36, 6.74%)	Eg.
		Induced abortion prescribed wrong as pregnant state
		Female infertility prescribed wrong as pregnant state
		Breast tumor prescribed wrong as breast mass
	Wrong route of administration (33, 6.18%)	Top 3
		External use prescribed wrong as oral use (4/33, 12.12%)
		Intramuscular injection prescribed wrong as intravenous injection (3/33, 9.09%)
		Oral use prescribed wrong as sublingual use (2/33, 6.06%)
	Wrong drug (10, 1.87%)	Top 3
		Low molecular weight heparin prescribed wrong as unfractionated heparin (2/10, 20%)
		Levofloxacin ear drops prescribed wrong as levofloxacin eye drops (1/10, 10%)
		Penicillin injection prescribed wrong as penicillin for skin test (1/10, 10%)
	Contraindication (6, 1.12%)	Top3
		Compound cold medication used under 2-year-old (4/6, 66.67%)
		Albendazole used under 2 years old (1/6, 16.67%)
		Ursodeoxycholic acid used during first trimester of pregnancy (1/6, 16.67%)
	Adverse drug-drug interaction (3, 0.56%)	Azithromycin powder diluted with wrong solvent 10% dextrose (2/3, 66.67%)
		Recombinant Human Growth Hormone diluted with wrong solvent 0.9% saline (1/3, 33.33%)
	Wrong strength (2, 0.37%)	Using 100 ml of 0.9% saline for intravenous bolus injection (1/2, 50%)
		Using 0.4 g/tablet of folic acid for treatment of anemia (1/2, 50%)
	Others (13, 2.43%)	Any error not falling into one of the above
Total	534 (100%)	

^a^ The missing diagnosis was recognized through the communication with physicians

^b^ Typing error was defined as error which was not caused by the physician’s intention, e.g. typing the wrong name of a drug which is similar to another drug, or typing the wrong measurement like “100 ml” as “10 ml” by mistake

^c^ Conscious act was defined as error caused by physician due to not updating the knowledge of related drug or prescribing an unfamiliar drug

^d^ ‘Bid’ refers to twice per day; ‘Qd’ refers to once per day; ‘Tid’ refers to thrice per day

**Fig. 2:**
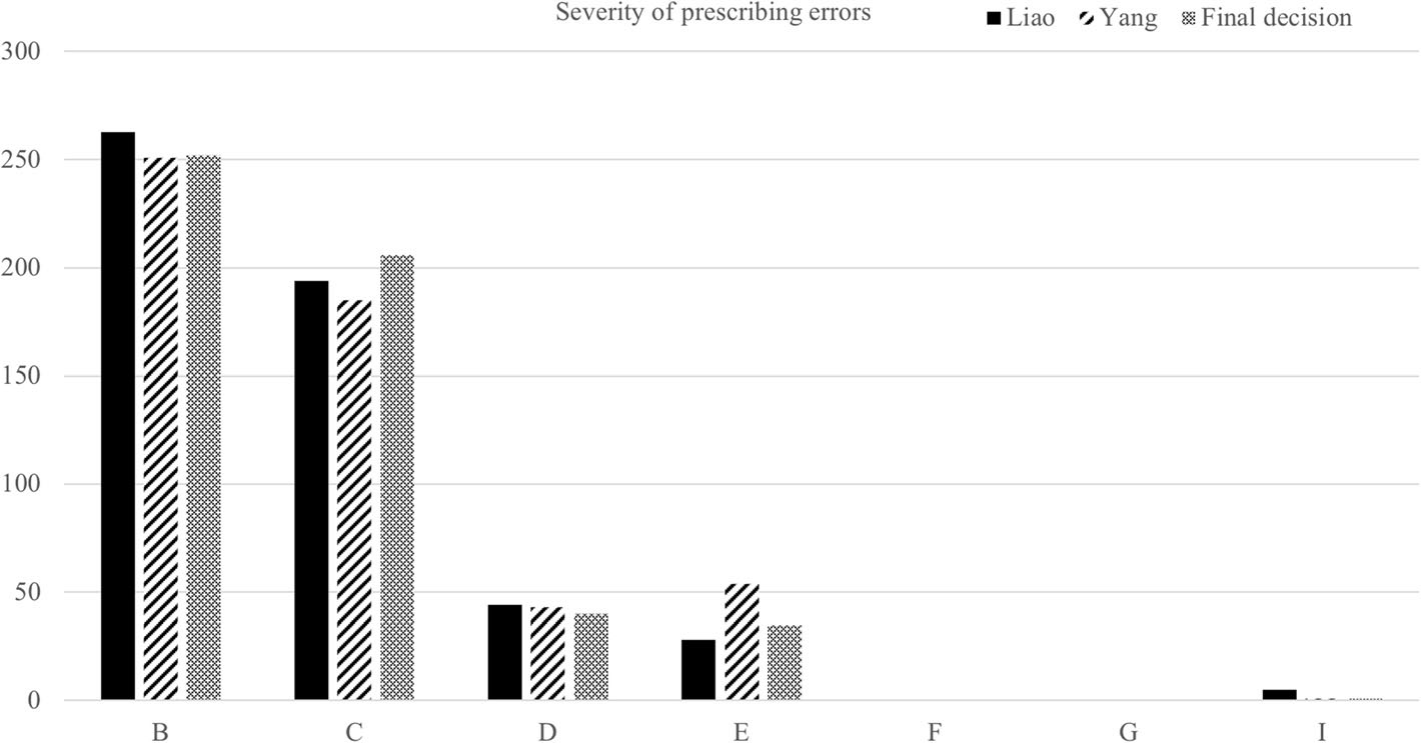
The assessment of the severity of prescribing errors by two investigators according to NCCMERP standards

The result of the assessment of error severity (by Yang and Liao) was shown in Fig. 2. A Kappa coefficient of 0.757 indicated a high level of agreement on assessment between investigators. There were 76 severe errors (severity level D-I), accounting for 14.23% of total prescribing errors (76 out of 534).

The output of likelihood ratio test from the logistic regression analysis gave a *P*-value less than 0.001, indicating the model fit well. The subgroup analysis of prescribing errors was shown at Table 2. The overall prescribing error rate was 0.34%. Prescribing more drug orders in one prescription was a potential risk factor as error rate tended to rise along with the increasing number of drug orders (between 1 and 5) in each prescription. Pediatric patients aged 29 days to 12 years were more likely to encounter prescribing errors, compared with neonates, adolescents and adults. Physicians prescribing on weekdays (0.39%) were more likely to make prescribing errors than on weekends.

**Table 2:** Subgroup analysis of rates of prescribing errors in each category

Category	Total	Prescriptions with error	Total error rates^a^	*P* value	OR [95% CI]
No. of drug orders per prescription^b^				0.001^c^	1.14 [1.06–1.23]
1	70,540	200	0.28%		
2	40,630	124	0.31%		
3	22,101	101	0.46%		
4	11,215	52	0.46%		
5	5401	32	0.59%		
6	564	1	0.18%		
7	101	0	0.00%		
8	31	0	0.00%		
9	23	0	0.00%		
10	5	0	0.00%		
Total	150,611	510	0.34%		
Age groups of patients				0.09^c^	
Neonate (~ 28 days)	6038	11	0.18%	Reference	
Infant (29 days to 12 months)	17,218	72	0.42%	0.01	2.33 [1.23–4.40]
Child (1 to 12 years)	41,292	166	0.40%	0.02	2.03 [1.10–3.76]
Adolescent (13 to 18 years)	700	2	0.29%	0.57	1.57 [0.34–7.36]
Adult (18 year~)	85,363	259	0.30%	0.42	1.43 [0.60–3.42]
Total	150,611	510	0.34%		
Seniority of physicians				0.31^c^	
Junior	11,960	36	0.30%	Reference	
Intermediate	52,621	155	0.29%	0.40	0.85 [0.58–1.24]
Senior	86,030	319	0.37%	0.98	0.99 [0.70–1.42]
Total	150,611	510	0.34%		
Specialty of physicians				< 0.001^c^	
Ophthalmology and otorhinolaryngology	5615	39	0.69%	Reference	
Family planning	13,421	75	0.56%	0.78	1.11 [0.55–2.26]
General surgery	4763	23	0.48%	0.76	0.89 [0.40–1.94]
Pediatrics	55,713	206	0.37%	< 0.001	0.45 [0.32–0.64]
General medicine	3807	13	0.34%	0.34	0.66 [0.28–1.55]
Reproductive medicine	12,634	38	0.30%	0.18	0.60 [0.28–1.27]
Gynecology	33,962	86	0.25%	0.04	0.49 [0.25–0.96]
Obstetrics	16,702	26	0.16%	0.003	0.31 [0.14–0.66]
Dermatology	3994	4	0.10%	< 0.001	0.13 [0.05–0.37]
Total	150,611	510	0.34%		
Work day				< 0.001^c^	
Weekends	30,534	72	0.24%	Reference	
Weekdays	120,077	438	0.36%	< 0.001	1.65 [1.27–2.14]
Total	150,611	510	0.34%		
Workload				0.97^c^	
Low	75,862	268	0.35%	Reference	
High	74,749	242	0.32%	0.97	1.00 [0.83–1.19]
Total	150,611	510	0.34%		

^a^ The rates were calculated by no. of prescriptions with errors by total prescriptions in each subgroup

^b^ The predictor of drug order was not taken into list box of categorical covariates at SPSS

^c^ Total *p* value of each predictor from likelihood ratio test

### The relationship between error rate and the prescribing workload of physicians

**Fig. 3:**
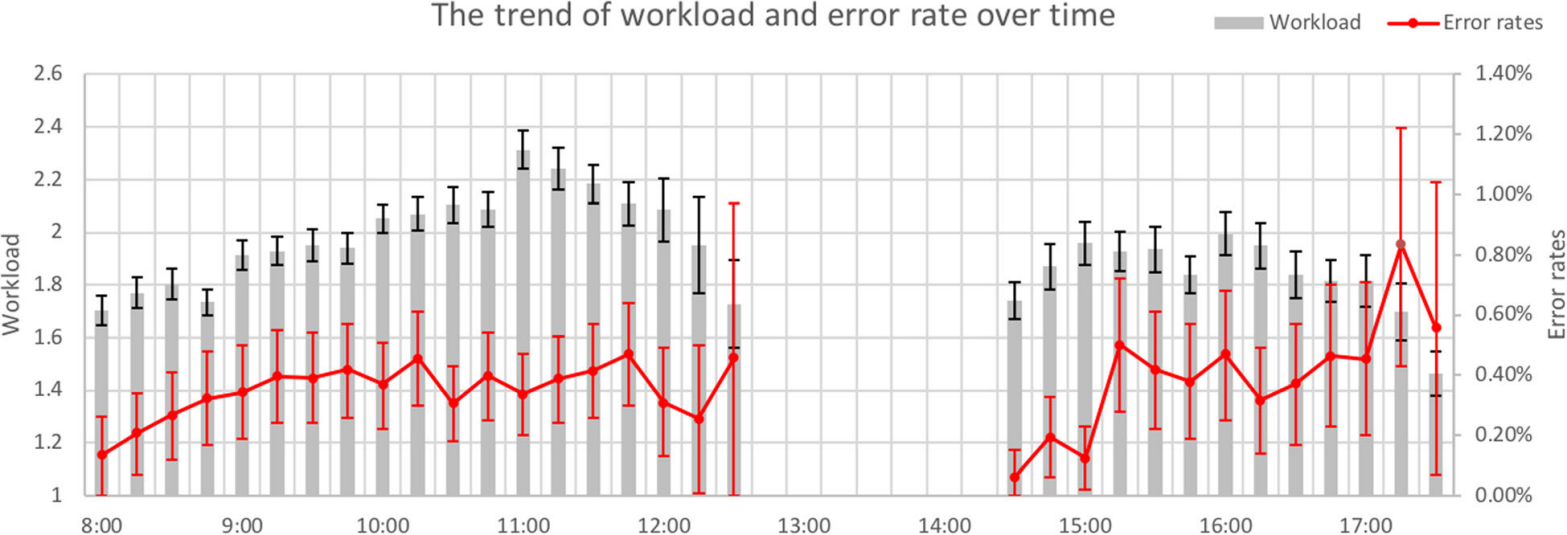
The trend of workload and error rate over time. Line graphs indicate the trend of error rate over time, while bar graphs indicate the workload trend

As shown in Fig. 3, the prescribing workloads increased over time from 8:00 to 11:00 and then decreased at the end of the morning shift. In the afternoon, the prescribing workloads maintained a plateau during 15:00–16:00 and fell down till the end of the afternoon shift. As to the error rate, it increased from 8:00 to 9:00 and fluctuated at a level of 0.40% between 9:00 and 12:00. The error rate presented an increasing tendency throughout the afternoon shift, reaching a peak between 17:15–17:30.

The autoregressive integrated moving average (ARIMA) model (*p* = 0, *d* = 2, *p* = 1) was selected in the time-series analysis. Stationary *R* squared value of 0.889 and Ljung-Box test (*Q* = 17.69, *P* > 0.05) indicated the acceptable goodness of fit of the model. The *β* value of independent variable (workload) was − 3.20*10^− 4^ (*P* > 0.05), indicating there was no correlation between workload and error rate.

## Discussion

### Overview

Pharmacist-led intervention was a common practice for hospitalized patients worldwide, such as in Brazil [27] and France [28], but seldom performed for outpatients. Our study showed that pharmacist-led intervention also played an important role in ensuring drug safety for outpatients. During a 3-month study period, pharmacists had intercepted more than 500 prescribing errors. Moreover, their interventions had made it to avoid 76 severe errors that may cause harm to patients.

Compared with other studies on handwritten prescribing [29, 30], our study on prescribing errors in e-prescriptions demonstrated a lower error rate of 0.34% (1.9 per 1000 orders). These results were similar to the review research by Weingart SN et al. [31] which found that errors occurred from 1.5 to 2 per 1000 orders with e-prescribing systems. On the contrary, Gilligan et al. [32] found that the difference between e-prescribing (11.7%) and handwritten (15.4%) prescribing, which might be caused by prescribers’ inexperience with the system in community pharmacy practice, was not statistically significant.

By using the e-prescribing system, absence of any prescription components including prescription date, clinic department, patient’s personal identifiers, drug regimen, physician’s electronic signature and the price of all drugs, would not occur due to an automatic intervention by the system, which contributed, to some extent, to a lower error rate than in the handwritten mode. In addition, the error type of wrong therapy duration included in other studies [32, 33] was excluded in ours because the medical records were unavailable from outpatients at pharmacies and a routine 7-day duration of drug therapy was prescribed at each outpatient visit in most cases. Moreover, the discrepancy in error rates could result from different designs of e-prescribing systems, as shown in a finding that the number, type, and severity of prescribing errors varied significantly based on the different computerized prescribing systems that were used, with prescribing error rates ranking from 5.1% to 37.5% [33].

### Potential risk factors of prescribing errors according to a subgroup analysis

Our study employed a subgroup analysis of prescribing errors according to the characteristic of drug therapy regimen, patients and physicians. Multiple orders in drug regimen, pediatric patients aged 29 days to 12 years, physicians specializing in ophthalmology and otorhinolaryngology, and prescribing on weekdays were found likely to be associated with prescribing errors.

Children were determined as a risk factor due to the multiple strengths of compounded medications and weight-based dosing which involved more calculations than for adults, so they were 3 times more likely to suffer from medication errors [34]. In our study, children aged 29 days to 12 years encountered more dosing errors than those of other age groups (not shown), which also validated this risk factor. The low error rate of prescriptions for neonates might stem from less types of diseases that neonates suffered in outpatient setting, with hyperbilirubinemia as the most common one. The dosing of most drugs for adolescents was similar to that for adults, so the error rate among adolescents was at a low level as well.

Our study found out that prescribing errors tended to increase along with the number of drug orders per prescription. The finding is consistent with studies [35, 36] which identified this variable as a significant risk factor. A systematic analysis [37] also indicated that the number of drug orders was the most common independent risk factor of errors resulting in serious adverse reactions.

Besides, our study figured out a decreasing trend of error rates from weekdays to weekends. This finding was similar to that of a study by Fijn R et al. [38]. However, the results of previous studies were [39–41] different from ours that the “weekend effect” tended to cause more errors. This difference might stem from the fact that the pharmacy staff working at weekends, including pharmacists from other departments, such as clinical pharmacists, were not representative of those usually working on weekdays. They were less familiar with the workflow of recording physicians’ prescribing errors and might accordingly underreport the errors. Given the combination of variable factors, this result should be regarded with caution.

### The relationship among error rate, prescribing workload and working time slot

Our study results challenged the general knowledge that a high prescribing workload will easily lead to errors [15, 16, 42], but they were similar to the finding of a study by Westbrook et al. [43] which showed that prescribing workload was in no close relation to errors rate. In another study [44], Fosbrook et al. demonstrated that prescribing errors were independent of prescribing workloads, and they proposed a hypothesis that there might be a relationship between prescription length and error rate since consultants with a low error rate frequently prescribed a single agent, which was verified by our subgroup analysis based on the number of drug orders in each prescription.

The existing study [45] demonstrating relationship between prescribing workload and error rate focused on the final results of statistical analyses, without involving time-specific trends of the above two variables. Our study, however, employed a time-series analysis not yet carried out, to our knowledge, in previous studies to illustrate the trend of prescribing workload and error rate over time. As shown in Fig. 3, during the first working hour (8:00–9:00) in the morning, the error rate and the prescribing workload both increased over time. The low error rate at the beginning of the shift might stem from the fact that physicians felt less tired and had a lower prescribing workload. Afterwards, the prescribing workload increased while the error rate maintained at a platform level of 0.4% with a maximum within 0.5%. It was assumed that there existed a threshold value exceeding which the prescribing workload, even during the busy time, would have little impact on the error rate.

It should be noted that the error rate increased over time even though the prescribing workload gradually decreased after 16:00 in the afternoon. Compared with the beginning of a day shift, the interesting finding at the end of the shift showed an increasing trend of error rate with decreasing prescribing workloads. Given the hypothesis that physicians were prepared in the final hour to leave on time to avoid traffic jams, relaxation and distraction were considered to be two key factors leading to their poor practice. This result was similar to that in a study by Vik et al. [46] in which patient care tended to suffer from more errors at the end of both 8-h and 12-h shifts than at the beginning.

## Limitations

There are several limitations in our study. As voluntary reporting systems are susceptible to under-reporting due to physicians’ unwillingness to report their own mistakes, the pharmacist-led reviewing mode applied in our study is also affected by under-reporting. The prescriptions with errors were identified by pharmacists in a stressful environment where their workload of reviewing prescriptions (60 prescriptions per hour) was high. Consequently, some errors were inevitably underreported and thus the error rates were underestimated. Furthermore, this study was carried out in an outpatient setting at only one special hospital. Accordingly, it should not be representative of other medical institutions.

## Conclusion

In summary, approximately 0.34% of prescriptions (1.9 per 1000 drug orders) with errors occurred during the 3-month study period at a tertiary women and children’s hospital, of which one in seven were classified as severe errors. Potential risk factors were figured out to include multiple drug orders, pediatric patients aged 29 days to 12 years, physicians specializing in ophthalmology and otorhinolaryngology and prescribing on weekdays. Moreover, no connection was found between error rate and prescribing workload. Nevertheless, further studies are needed to investigate pharmacist-led intervention to reduce prescribing errors.

## Data Availability

The datasets used and/or analyzed by the current study are available from the corresponding author on reasonable request.

## Abbreviations

ADEs: Adverse drugs events
ARIMA: Autoregressive integrated moving average
ATC: Anatomical therapeutic chemical
HIS: Hospital information system
NCC MERP: National coordinating council for medication error reporting and prevention
